# The mediating role of self-efficacy in the relationship between eHealth literacy and childbirth readiness among pregnant women: a cross-sectional study

**DOI:** 10.3389/fpubh.2025.1561855

**Published:** 2025-04-09

**Authors:** Ningying Zhou, Danni Wu, Min Liu, Shanshan Hu, Feng Zhang, Yuqing Zan, Fei Sun

**Affiliations:** ^1^Wuxi School of Medicine, Jiangnan University, Wuxi, Jiangsu, China; ^2^Department of Nursing, Affiliated Women's Hospital of Jiangnan University, Wuxi, Jiangsu, China; ^3^Department of Obstetrical, Affiliated Hangzhou First People's Hospital, School of Medicine, Westlake University, Hangzhou, China

**Keywords:** pregnant women, childbirth readiness, eHealth literacy, self-efficacy, mediating effect

## Abstract

**Background:**

Childbirth readiness is an important component of maternal and child health. Therefore, it is imperative to understand identify the factors influencing childbirth readiness to develop interventions for improving women's wellbeing. In the current digital age, it is crucial to explore the link between eHealth literacy and childbirth readiness. However, few studies have investigated this relationship. Here, we explored the effect of eHealth literacy on childbirth readiness and examined the mediating role of self-efficacy in this relationship.

**Methods:**

A total of 350 third-trimester pregnant women were enrolled in this survey from April to June 2023 at the outpatient departments of the Affiliated Women's Hospital of Jiangnan University, China. Four questionnaires were used to collect data from participants: the General Information Questionnaire, eHealth Literacy Scale (eHEALS), General Self-Efficacy Scale (GSES), and Childbirth Readiness Scale (CRS). The associations among variables were determined through Pearson correlation analysis, and the Amos 26.0 software was employed to analyze the mediating role of self-efficacy.

**Results:**

A total of 350 surveys were distributed to the participants, and 338 eligible questionnaires were finally collected, translating to a response rate of 96.57%. The average score of participants was 27.39 ± 6.40 on the GSES, 32.15 ± 6.16 on the eHEALS, and 74.26 ± 8.81 on the CRS. The eHEALS scores exhibited a strong positive association with self-efficacy (*r* = 0.509, *P* < 0.01), which in turn was positively correlated with childbirth readiness (*r* = 0.505, *P* < 0.01). Self-efficacy mediated the relationship between eHealth literacy and childbirth readiness, accounting for 33.02% of the total effect.

**Conclusions:**

E-health literacy is a positive predictor of childbirth readiness among pregnant women, with self-efficacy mediating the association. These findings provide a basis for developing targeted interventions to improve childbirth readiness.

## 1 Introduction

Pregnancy and childbirth induce several physical changes, role conflicts, and psychological emotional transformations in women (1, 2). Moreover, these effects are unpredictable, posing significant challenges for women. According to the World Health Organization, a woman dies every 2 min during pregnancy or childbirth worldwide (3). The Millennium Development Goals and the Global Strategy for Women's, Children's, and Adolescents' Health advocate for interventions for reducing maternal mortality, enhancing the experience of childbirth for women, and provision of high-quality care (4). Evidence from prior investigations show that maternal and newborn deaths can be prevented through implementation of effective strategies targeting to enhance preparedness for childbirth (5).

Childbirth readiness encompasses the collective assessments for various capabilities of pregnant women prior to childbirth, covering material preparedness, knowledge acquisition, psychological readiness, planning, and management skills. It is used as an indicator for predicting women's ability to cope with childbirth behavior during the delivery process (6, 7). Sufficient childbirth readiness can reduce maternal and neonatal mortality, present occurrence of pregnancy-related complications, and improve the childbirth experience (8, 9). In contrast, inadequate childbirth readiness arouses negative emotions such as fear of childbirth and pregnancy anxiety, which can affect the mother-child relationship and even a woman's desire to have children (10, 11). To protect the interests of women and children, WHO has included childbirth readiness as an important component of antenatal care (12).

To date, it has been reported that the level of childbirth readiness various significantly among pregnant women with respect to regions and racial groups (13, 14). These disparities are induced by diverse factors specific to certain vulnerable populations, such as transportation limitations and economic constraints, which limit accessibility to prenatal counseling and services (15). Furthermore, the physical burden imposed by fetal growth and development during pregnancy restricts women's mobility, which restricts their chances of accessing prenatal care (16). The other challenging affecting pregnant women in developing countries is the shortage of healthcare professionals and prolonged waiting times, which decreases their capacity to acquire childbirth readiness-related knowledge (17).

Considering these challenges, electronic resources have become important sources of information regarding childbirth. Data shows that 74% of China's population used internet resources in 2022 (18), suggesting that the Internet is likely to be an important source of health information for pregnant women (19). The Internet can avoid the limitations associated with time and space. For example, during the COVID-19 pandemic in China, pregnant women exhibited a preference for online courses and information regarding pregnancy and childbirth to reduce the risk of infection from hospital visits (20). In addition, electronic resources available on the Internet are cheap, convenient, open, and diversification (21, 22). This indicates that the Internet, as a powerful platform, offers new solutions to medical service challenges caused by geographical gaps, economic difficulties, and resource shortages (23). It opens up new ideas for improving childbirth readiness.

However, the successful utilization of electronic resources is dependent on an individuals' electronic health literacy. E-health literacy is concept that refers to an individual's capacity to search for, understand, and critically evaluate health-related information from digital sources, and to apply the acquired knowledge to address or manage health concerns (24). People with high eHealth literacy can utilize and benefit more from health knowledge (25). Thus, eHealth literacy is an important factor influencing healthy behavior, for example, eHealth literacy can effectively improve individual self-management (26, 27). In the context of childbirth readiness, multiple components should be considered, with self-management being a part of them (7). Therefore, researchers need to investigate the relationship between eHealth literacy and childbirth readiness to identify bottlenecks and develop interventions.

Previous studies have demonstrated a positive correlation between eHealth literacy and self-efficacy (28). Self-efficacy refers to people's belief in completing a task or performing a certain behavior despite the prevailing life challenges (29). People with high eHealth literacy capable of utilizing electronic resources possess higher knowledge levels, but also confidence in adopting healthy behaviors (30). Self-efficacy has been shown to potentially alleviate negative emotions and enhance the ability of individuals to cope with pregnancy stress in postpartum (31). Pregnant women with high self-efficacy are more confident and can withstand pregnancy difficulties and approach childbirth readiness with a more positive attitude (32). Majority of previous investigations focused on the pairwise relationships between eHealth literacy, self-efficacy, and childbirth readiness, but no literature has explored the potential interconnections among all three factors.

In this study, we employed the knowledge-attitude-behavior theory, which is commonly used in the health promotion (33). It divides human behavior change into three stages: acquiring knowledge, forming beliefs, and taking action. This theory states that knowledge is the cornerstone of changing behavior, belief is its intrinsic driving force, and behavior is the ultimate practical goal (34). In this study, we defined knowledge level corresponding to eHealth literacy refers as the ability of pregnant women to acquire and utilize pregnancy and childbirth knowledge through electronic resources. In previous studies, self-efficacy was employed to measure the personal beliefs (29), which affects their practical behavior before delivery, referred to as childbirth readiness. In addition, self-efficacy has been proposed to mediate the relationship between eHealth literacy and health promoting behavior in older adults, demonstrating that this theory can be applied in promoting health behavior (30). Therefore, we adopted the knowledge-attitude-behavior theory to construct a structural equation model (SEM) which was applied to dissect the relationships among eHealth literacy, self-efficacy, and childbirth readiness. The results of this study providing a theoretical basis for taking childbirth readiness-related interventions and promoting maternal and child health. This study proposes the following hypothesis: (1) eHealth literacy is positively associated with childbirth readiness; (2) eHealth literacy indirectly influences childbirth readiness through enhanced self-efficacy.

## 2 Materials and methods

### 2.1 Ethical recognition

The study approved by the Institutional Ethics Committee of Affiliated Women's Hospital of Jiangnan University (No 2021-01-1215-32, in 2021).

### 2.2 Study design and participant

A cross-sectional design was adopted in this study. A convenience sampling method was adopted to select outpatient pregnant women in the third trimester from April to June 2023 at Affiliated Women's Hospital of Jiangnan University, Jiangsu Province, China. The following criteria were utilized to enroll participants: (1) age ≥18 years; (2) voluntary participation with signed informed consent; and (3) gestational week ≥28 weeks. Those who met the following criteria were excluded from the study: (1) inability to complete the questionnaire; (2) severe mental illness; (3) serious pregnancy-related comorbidities or complications; and (4) indications for cesarean section. To meet the recommended sample size for general structural equation modeling of ≥200, we enrolled 350 pregnant women as study subjects (35).

### 2.3 Data collection

**Figure 1:**
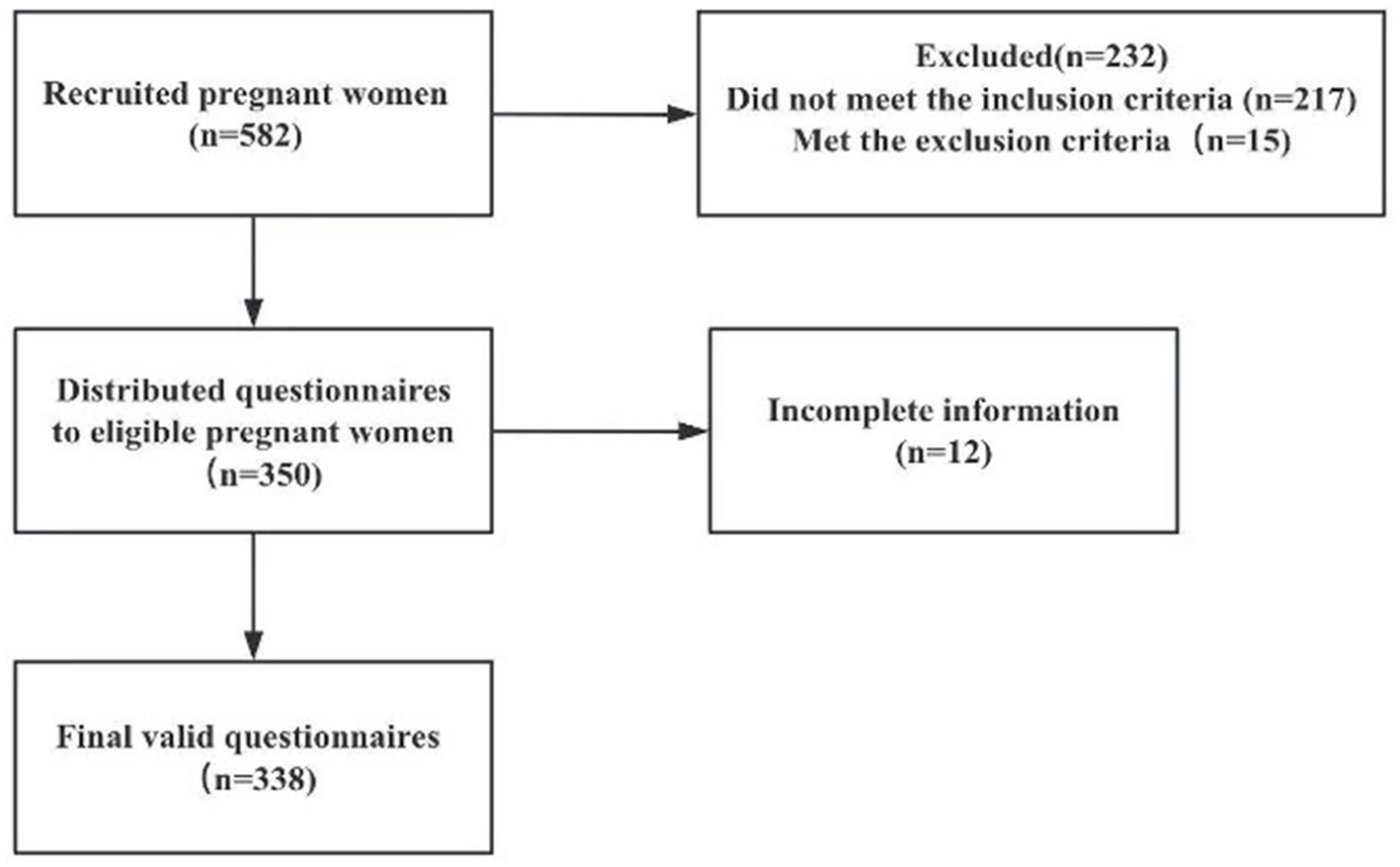
Flow diagram of participant recruitment (pregnant women).

A survey team comprising three members, trained uniformly, conducted the questionnaire survey to ensure consistent terminology, and full understanding of the scale. The face-to-face survey was voluntary, and participants were informed of the study's purpose and significance beforehand. Participants were assured of their right to withdraw at any time, and their privacy was fully protected. Questionnaires were distributed on-site, completed independently by participants, checked for completeness by researchers, and collected immediately. In total, 350 questionnaires were distributed. Among these, 338 were deemed valid, corresponding to a response rate of 96.57%. The study recruitment flow chart is shown in Figure 1.

### 2.4 Instrument

#### 2.4.1 General demographic information questionnaire

The researchers developed the questionnaire based on a review of relevant literature. It included items on the pregnant woman's age, place of residence, educational level, use of pregnancy-related apps, parity, presence of pregnancy complications or comorbidities, and participation in prenatal classes.

#### 2.4.2 eHealth literacy

eHealth Literacy Scale (eHEALS), developed by Norman in 2006, is often used to evaluate an individuals' ability to seek, evaluate, and apply electronic health information (24). It was later adapted into Chinese by scholar Guo (36). The scale contains 8 items measuring three dimensions: application of online health information and services, critical evaluation skills, and decision-making abilities. Each item is scored on a 5-point Likert scale, from 1 (strongly disagree) to 5 (strongly agree), with overall scores ranging from 8 to 40. Higher scores indicate better eHealth literacy. In this study, the Cronbach's α was 0.935, demonstrating strong internal reliability.

#### 2.4.3 Self-efficacy

General Self-Efficacy Scale (GSES): Created by Schwarzer, the GSES comprises 10 items (37). Each item is assessed using a 4-point Likert scale, ranging from 1 (completely incorrect) to 4 (completely correct), with total scores ranging from 10 to 40. Higher scores indicate greater self-efficacy. The Cronbach's α coefficient in this study was 0.929.

#### 2.4.4 Childbirth readiness

Childbirth Readiness Scale (CRS): Developed by Chinese scholars Yuan Mengmei et al., comprises 18 items across four dimensions: self-management, information literacy, childbirth confidence, and childbirth planning. Items are rated on a 5-point scale, from 1 for strongly disagree to 5 for strongly agree, with possible total scores between 18 and 90. Lower scores indicate lesser childbirth readiness (7). The study found a Cronbach's α of 0.909, highlighting high internal consistency.

### 2.5 Statistical analysis

All data were analyzed using SPSS version 26.0. Categorical data were presented as frequencies and percentages (%) while continuous data that followed a normal distribution were described using means ± standard deviations. Group comparisons were conducted using independent samples *t*-tests or one-way ANOVA. Pearson correlation analysis was employed to explore the associations between eHealth literacy, childbirth readiness, and self-efficacy. Amos 26.0 will investigate the relationships between eHealth literacy, self-efficacy, and childbirth readiness. Bootstrap methods with 5,000 resamples were used to test mediation effects, with a 95% confidence interval that did not include zero indicating significant mediation. Model fit indices were evaluated based on the following criteria: the ratio of chi-square to degrees of freedom (χ^2^/df), root mean square error of approximation (RMSEA), goodness-of-fit index (GFI), adjusted goodness-of-fit index (AGFI), normed fit index (NFI), Tucker-Lewis index (TLI), comparative fit index (CFI), and incremental fit index (IFI). Statistical significance was set at *P* < 0.05.

## 3 Results

### 3.1 Respondent demographics and obstetric characteristics

A total of 338 pregnant women aged 18 to 41 years, with a mean age of 30.09 ± 3.88 years were enrolled in the study. Among the participants, 76.9% had an education level of college or above, 86.1% lived in county or urban areas, and 93.2% used pregnancy-related apps. Additionally, 67.2% were primigravid (first-time pregnant), 82.2% did not have any pregnancy comorbidities or complications, and 32.5% participated in maternity classes. There were significant differences in parity, presence of pregnancy comorbidities or complications, and participation in prenatal classes among the groups (*P* < 0.05; Table 1).

**Table 1:** Comparison of demographic and obstetric characteristics and childbirth readiness scores of pregnant women (n = 338).

**Variables**	***n* (%)**	**Childbirth readiness (Mean ±SD)**	**t/*F***	***P***
Age (years)			1.208	0.300
18–24	20 (5.9)	71.50 ± 9.86		
25–34	270 (79.9)	74.56 ± 8.61		
≥35	48 (14.2)	73.77 ± 9.48		
Education level			0.561	0.641
Junior high school or below	35 (10.4)	75.09 ± 7.69		
Senior high school/vocational school	43 (12.7)	73.33 ± 10.28		
College/bachelor's degree	239 (70.7)	74.15 ± 8.79		
Graduate degree or above	21 (6.2)	76.05 ± 7.80		
Place of residence			1.643	0.195
Rural area	47 (13.9)	73.38 ± 8.82		
County district	219 (64.8)	74.90 ± 8.24		
Urban area	72 (21.3)	72.92 ± 10.30		
Use of pregnancy-related apps			1.031	0.303
Yes	315 (93.2)	74.40 ± 8.79		
No	23 (6.8)	72.43 ± 9.20		
Parity			−5.149	<0.001
Primigravid	227 (67.2)	72.60 ± 8.40		
Multigravid	111 (32.8)	77.67 ± 8.70		
Pregnancy comorbidities or complications			−2.156	0.032
Yes	60 (17.8)	72.05 ± 7.70		
No	278 (82.2)	74.74 ± 8.98		
Participation in prenatal classes			3.205	0.001
Yes	110 (32.5)	76.45 ± 7.72		
No	228 (67.5)	73.21 ± 9.13		

### 3.2 Mean scores for eHealth literacy, childbirth readiness, and self-efficacy among pregnant women

The mean eHealth literacy, self-efficacy, and childbirth readiness scores for pregnant women were 32.15 ± 6.16, 27.39 ± 6.40, and 74.26 ± 8.81, respectively. The mean scores of the dimensions and entries of the scale are shown in Table 2.

**Table 2:** eHealth literacy, self-efficacy, and childbirth readiness scores of pregnant women (*n* = 338).

**Variables**	**Items**	**Total score**	**Mean item score**
eHEALS	8	32.15 ± 6.16	4.02 ± 0.77
Applied skills	5	20.69 ± 3.76	4.14 ± 0.75
Critical skills	2	7.70 ± 1.90	3.85 ± 0.95
Decision-making ability	1	3.75 ± 1.03	3.75 ± 1.03
GSES	10	27.39 ± 6.40	2.74 ± 0.64
CRS	18	74.26 ± 8.81	4.13 ± 0.49
Self-management	4	18.57 ± 1.60	4.64 ± 0.40
Information literacy	6	22.35 ± 4.45	3.73 ± 0.74
Childbirth confidence	4	15.94 ± 2.70	3.99 ± 0.68
Childbirth planning	4	17.40 ± 2.29	4.35 ± 0.57

GSES, General Self-Efficacy Scale; CRS, Childbirth Readiness Scale; eHEALS, eHealth Literacy Scale.

### 3.3 Correlation analysis between eHealth literacy, self-efficacy, and childbirth preparedness in pregnant women

The Pearson correlation analysis results showed a positive correlation between eHealth literacy and self-efficacy (*r* = 0.509, *P* < 0.01). A positive correlation existed between eHealth literacy and childbirth preparedness (*r* = 0.487, *P* < 0.01). It was also observed that self-efficacy was positively correlated with childbirth preparedness (*r* = 0.505, *P* < 0.01). Full details of the results are shown in Table 3.

**Table 3:** Pearson correlation analysis of self-efficacy, eHealth literacy, and childbirth readiness among pregnant women (*n* = 338).

**Variables**	**1**	**2**	**3**	**4**	**5**	**6**	**7**	**8**	**9**	**10**
1. CSES	1.000	–	–	–	–	–	–	–	–	–
2. eHEALS	0.509^**^	1.000	–	–	–	–	–	–	–	–
3. Applied Skills	0.456^**^	0.955^**^	1.000	–	–	–	–	–	–	–
4. Critical skills	0.468^**^	0.893^**^	0.735^**^	1.000	–	–	–	–	–	–
5. Decision-making ability	0.513^**^	0.847^**^	0.705^**^	0.811^**^	1.000	–	–	–	–	–
6. CRS	0.505^**^	0.487^**^	0.444^**^	0.438^**^	0.481^**^	1.000	–	–	–	–
7. Self-management	0.292^**^	0.330^**^	0.319^**^	0.282^**^	0.291^**^	0.567^**^	1.000	–	–	–
8. Information literacy	0.409^**^	0.440^**^	0.382^**^	0.430^**^	0.448^**^	0.884^**^	0.315^**^	1.000	–	–
9. Childbirth confidence	0.438^**^	0.384^**^	0.363^**^	0.309^**^	0.399^**^	0.815^**^	0.336^**^	0.611^**^	1.000	–
10. Childbirth planning	0.428^**^	0.335^**^	0.318^**^	0.291^**^	0.305^**^	0.774^**^	0.477^**^	0.522^**^	0.538^**^	1.000

** *P* < 0.01.

GSES, General Self-Efficacy Scale; CRS, Childbirth Readiness Scale; eHEALS, eHealth Literacy Scale.

**Figure 2:**
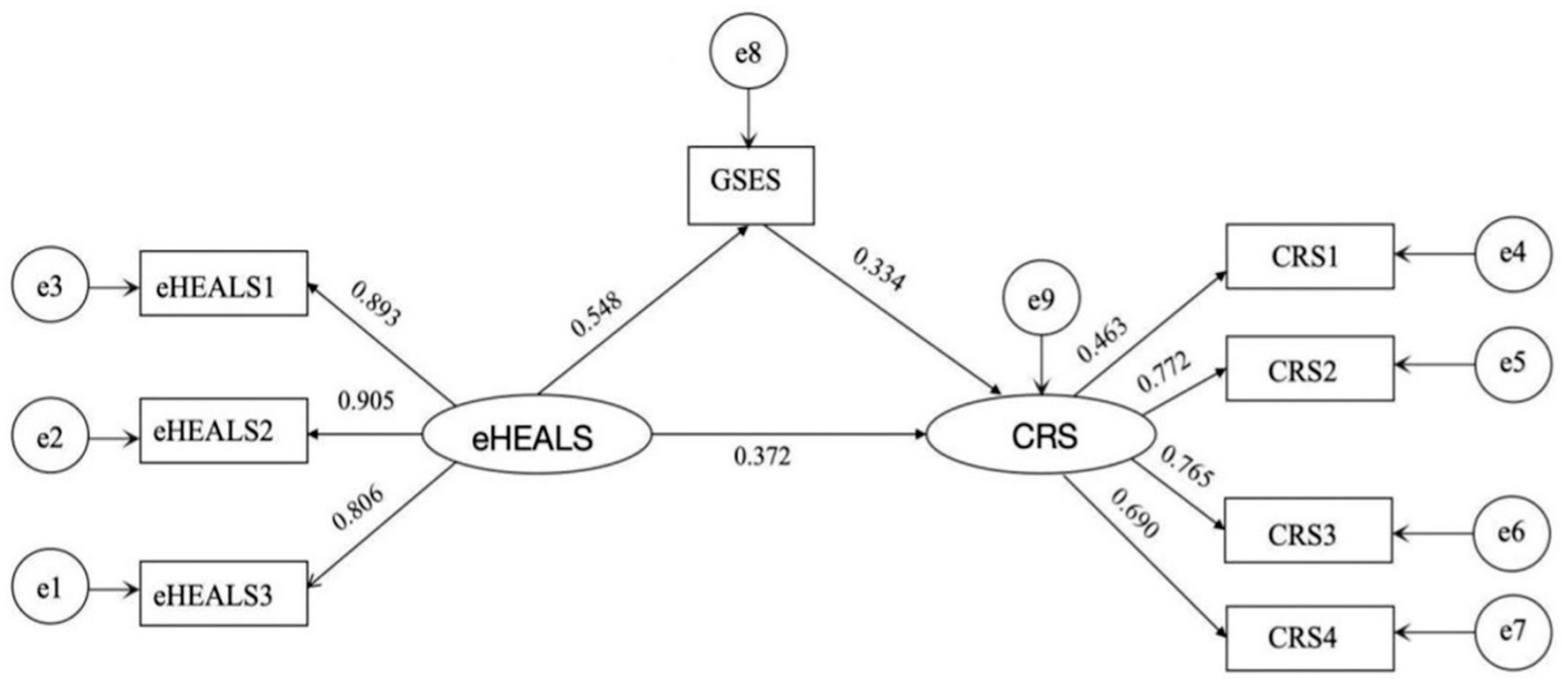
SEM of eHealth literacy, childbirth readiness, and self-efficacy constructs.

Correlation analysis identified pairwise correlations between eHealth literacy, self-efficacy, and childbirth readiness. A structural equation model (SEM) was constructed using Amos 26.0 software to further explore the relationships among these three variables. In this SEM, we controlled for factors that were significant in the univariate analyses, including parity, pregnancy complications or comorbidities, and prenatal class attendance. The model was established using the eHealth literacy as the independent variable, self-efficacy as the mediating variable, and childbirth readiness as the dependent variable. The standardized path coefficients of the model are shown in Figure 2. Model fit indices were assessed, and all met the required standards (Table 4), indicating a good model fit.

**Table 4:** Structural equation fitting index.

**Index**	**χ^2^/df**	**RMSEA**	**GFI**	**NFI**	**IFI**	**TLI**	**CFI**
Result	2.834	0.074	0.936	0.916	0.944	0.926	0.943
Standard value	<3.000	<0.080	>0.900	>0.900	>0.900	>0.900	>0.900

χ^2^/df, the ratio of chi-square to degrees of freedom; RMSEA, root mean square error of approximation; GFI, goodness-of-fit index; NFI, normed fit index; TLI, Tucker-Lewis index; CFI, comparative fit index; IFI incremental fit index.

The Bootstrap method was used to perform 5,000 random resamples from the original dataset. The analysis revealed that the 95% confidence intervals for the direct, indirect, and mediating effects did not include zero. This result suggests that self-efficacy partially mediates the relationship between eHealth literacy and childbirth readiness. Specifically, the total effect of eHealth literacy on childbirth readiness was 0.318, while the direct effect of eHealth literacy on childbirth readiness was 0.213, and the indirect effect through self-efficacy was 0.105. This indicates that self-efficacy partially mediates the relationship, accounting for ~33.02% of the total effect (as detailed in Table 5).

**Table 5:** Analysis of the mediating effect of self-efficacy between eHealth literacy and childbirth readiness.

**Effect**	**Effect size**	**Standard error**	***P***	**95% CI**	**Relative effect ratio (%)**
Total effect	0.318	0.054	<0.001	0.223–0.437	**–**
Direct effect	0.213	0.051	<0.001	0.127–0.328	66.98
Indirect effect	0.105	0.028	<0.001	0.058–0.170	33.02

## 4 Discussion

In this study, investigated that relationships among eHealth literacy, childbirth readiness, and self-efficacy in pregnant women and the mediating role of self-efficacy in the relationship between eHealth literacy and childbirth readiness. The study uncovered the potential pathways linking eHealth literacy to childbirth readiness.

The results indicated that the eHEALS score for pregnant women was 32.15 ± 6.16, indicating a medium to high level of eHealth literacy. This score is higher than that reported by Rahdar et al. (38). The difference may be arising from variations among geographic factors. Our participants were from the Yangtze River Delta, China's most economically active region. Here, per capita income and socioeconomic levels are higher compared to the Zahedan region in Iran. Given that the eHealth literacy may be affected by income and socioeconomic factors, it is not surprising that the eHealth literacy levels in this study were higher (39). In addition, among all dimensions of eHealth literacy, the decision-making dimension scored lower than the others, similar to CHAO's findings (40). While the digital age has increased access to health information and online resources for pregnant women, several challenges remain. A previous qualitative investigation reported that pregnant women with gestational diabetes mellitus may receive contradictory advice from information derived from electronic resources, which can hinder their decision-making (41). Moreover, the quality of online information varies, which reduces people's confidence in its safety, source, and reliability. This limits the application of health information to improve health behaviors (40). These findings suggest that healthcare organizations should promote the creation of online mutual support platforms for doctors and patients. Such platforms can provide safe, accurate, and high-quality health resources for pregnant women.

Data analysis revealed that pregnant women's GSES score was 27.39 ± 6.40, indicating an upper-middle level of self-efficacy, similar to Bień's findings (42). This may be due to higher education levels, better family economic status, and more participation in prenatal classes. Higher education helps women understand and adopt new knowledge and skills. Better economic status provides more support resources. Prenatal classes provided important health information which reduced pregnant women's negative emotions (10). Factors like education, income, health education, and emotional state all impact self-efficacy (43, 44). Thus, improving support systems through family and community efforts can enhance self-efficacy and boost confidence in managing pregnancy challenges.

In this found, we found that the CRS score for pregnant women was 74.26 ± 8.81, implying an upper-middle level of childbirth readiness, though lower than that reported by Zeng et al. (11). This difference may be explained by the inclusion of women with smaller gestational weeks in this study. According to China's maternity policy, women with larger gestational weeks typically have more antenatal care visits, which can increase awareness of childbirth danger signs and improve childbirth readiness (45). Therefore, antenatal care's frequency, quality, and content adequacy are crucial and warrant attention from health workers.

Interestingly, 92.3% of pregnant women in this study used pregnancy apps, yet the childbirth readiness scores did not show a large difference. Pregnancy apps can serve as valuable electronic resources. However, Nissen reported that only a few apps involve health professionals in their development (46). Inaccurate information, contradictory advice, excessive commercial advertisements, and the lack of comprehensive pregnancy-related content in these apps may undermine pregnant women's trust and negatively impact their user experience (47). This could hinder improvements in their childbirth readiness. Therefore, government departments should encourage healthcare professionals to collaborate with pregnancy application developers to create scientifically validated, comprehensive, and user-friendly products. Additionally, healthcare providers can assist pregnant women in identifying reliable applications through standardized rating tools and offer detailed guidance on their features, enabling pregnant women to fully utilize these electronic resources.

This study demonstrated a positive correlation between eHealth literacy and childbirth readiness among pregnant women (*r* = 0.487, *P* < 0.01). Structural equation modeling revealed that higher eHealth literacy directly predicts better childbirth readiness. Ahmed found that social media access and use significantly influence childbirth readiness (48). Pregnant women with higher eHealth literacy are more likely to use social media for pregnancy-related information and support, as noted by George et al. (49). Pregnant women can utilize electronic resources to learn about fetal development, manage pregnancy symptoms, and familiarize themselves with the labor process. High eHealth literacy also promotes sharing pregnancy and childbirth experiences, reducing stress, and anxiety through peer communication (41). Therefore, healthcare professionals need to formulate simple, user-friendly online platforms that can improve pregnant women's capacity to identify reliable information and improve their decision-making skills. Enhancing eHealth literacy in this way can boost overall childbirth readiness.

The present result showed that eHealth literacy levels of pregnant women were positively associated with self-efficacy, which is consistent with the reports by Lee et al.'s (26) who investigated diabetic patients. Pregnant women with higher eHealth literacy can access quality information and learn pregnancy-related knowledge and skills, which reduces their uncertainty about upcoming pregnancy events. Lower intolerance of uncertainty is linked to higher self-efficacy (50).

The findings of this study support the hypothesis that self-efficacy partially mediates the relationship between maternal eHealth literacy and childbirth readiness. Notably, the indirect effect of eHealth literacy on childbirth readiness through self-efficacy accounted for 32.89% of the total effect (*P* < 0.01). eHealth literacy not only directly predict the level of childbirth readiness but also indirectly influence childbirth readiness through self-efficacy. According to the knowledge, attitude, and behavior theory, self-efficacy plays a crucial role as a bridge connecting knowledge and action (30). Individuals with high-level electronic health literacy may acquire, understand, and utilize more knowledge (25). High knowledge levels enhance pregnant women's confidence in overcoming challenges during pregnancy, encourages active engagement in healthy behaviors such as self-management during pregnancy and the development of birth plans, and thereby promotes the improvement of childbirth readiness (51). This suggests that promoting healthy behaviors requires sufficient knowledge and attention to psychological factors such as self-efficacy (52). Research has shown that interventions related to mindfulness education and empowerment education can help improve the self-efficacy of pregnant women (53). Therefore, healthcare providers should not only focus on assessing pregnant women's electronic health literacy levels but also implement proactive interventions to enhance their self-efficacy, ultimately promoting the improvement of childbirth readiness.

## 5 Conclusions

This study demonstrates that eHealth literacy and self-efficacy of pregnant women are positively associated with childbirth readiness. Self-efficacy partially mediates the association between e-health literacy and childbirth readiness. These findings are expected to guide the development of interventions to promote childbirth readiness among pregnant women. On the one hand, they show that healthcare workers should aim to enhance the eHealth literacy level of pregnant women by developing strategies to increase decision-making skills. Moreover, they should encourage the utilization of e-resources, screening, and development of high-quality pregnancy apps. During the design of childbirth readiness interventions, healthcare professionals should focus on enhancing pregnant women's self-efficacy while improving e-health literacy, thus improving childbirth readiness.

## 6 Limitations

Firstly, this study employed convenience sampling and was conducted at a single center, which may limit the representativeness of the sample. Future research should adopt a multi-center randomized sampling approach to enhance the generalizability of the findings. Secondly, all participants in this study were women in late pregnancy, which may influence the outcomes related to childbirth readiness. In future studies, pregnant women at various gestational stages should be included. Lastly, as this study is cross-sectional, it does not allow for the determination of causal relationships between variables. Future research could adopt a longitudinal study design to investigate these relationships further.

## Data Availability

The original contributions presented in the study are included in the article/Supplementary material, further inquiries can be directed to the corresponding author/s.
